# Mindfulness Matters: Untangling Entrapment and Mental Well-Being in Married Couples

**DOI:** 10.1007/s11126-025-10168-0

**Published:** 2025-06-11

**Authors:** Yusuf Akyıl, Beste Erdinç, Seydi Ahmet Satıcı

**Affiliations:** 1 Department of Psychological Counseling, Ministry of Education, Istanbul, Türkiye; 2 Department of Psychological Counseling, Yıldız Technical University, Istanbul, Türkiye

**Keywords:** Entrapment, Mindfulness, Mental well-being, Actor-partner interdependence model, Dyadic

## Abstract

Individual experiences can influence the mental health of married couples. The objective of the present study is to investigate the mediating role of mindfulness in marriage between entrapment and mental well-being by examining the relationships between actors and partners. The actor-partner interdependence mediation model was employed to apply a dyadic approach to the data collected from 423 (*N = *846) married couples for this purpose. The mean age of the women was 35.80 (SD = 9.57), while the mean age of the men was 39.0 (SD = 10.46). The results indicated that the relationships between entrapment, mindfulness, and mental well-being were substantial for both genders. It was discovered that the entrapment experienced by each of the couples predicted their own well-being through the mindfulness of the other in marriage in the context of partner effects. In other words, women's entrapment predicted their mental well-being, which in turn predicted men's mindfulness in marriage. Similarly, the mindfulness of women in marriage predicts the mental well-being of men. Consequently, it is anticipated that the well-being of both spouses will be enhanced if the mindfulness levels of individuals experiencing entrapment are high.

## Introduction

Marriage serves as the foundational institution establishing the family unit and plays a crucial role in societal cohesion. However, recent demographic trends reveal a decline in marriage rates and an increase in divorce rates; for instance, the European Union has experienced a decrease in the number of marriages per capita alongside a rise in divorces [1]. The United States had 62.18 million married spouses in 2023 [2]. It is imperative to prioritize the enhancement of marital relationships in order to foster the well-being of future generations and to establish a harmonious family and society. In addition to individual attention, it is crucial to address the mental health of married couples for this purpose. Married individuals'well-being, marital satisfaction, self-compassion, and happiness-enhancing strategies are known to significantly correlate [3].

An individual's capacity to establish and sustain loving relationships with others, fulfill social roles commonly played in their culture, manage change, recognize, accept, and communicate positive actions and thoughts, and manage negative emotions such as sadness is known as mental well-being. Mental health provides the individual with a sense of worth, control, and comprehension of internal and external functioning [4]. Tennant et al. [5] interchangeably refer to mental well-being as positive mental health. Well-being is both the hedonistic experience of feeling happy and the eudaimonic experience of fulfillment and purpose that an individual has. It is capable of fluctuating over the course of weeks, days, and even hours, as well as changing over time [6]. Individuals who have positive relationships have superior mental health compared to those who have negative relationships or no relationships at all [7]. Concurrently, marital satisfaction and conflict resolution are positively correlated with well-being (Ali et al., 2024).

The Systemic Transactional Model (STM) strongly intertwines partners'pleasure and well-being. Consequently, the STM underscores the importance of reciprocity and interdependence among companions. Consequently, the happiness and stress of one companion consistently influence the other [8]. There are numerous methods by which individuals can react to tension. Involuntary Defeat Strategy (IDS) is a method that was devised to elucidate this phenomenon. The method posits that stress, operating between defeat perception and entrapment, can lead to depression, anxiety, and PTSD. Entrapment, characterized by a persistently overactive IDS response, establishes a maladaptive or"depressogenic feedback loop"in individuals [9]. Defeat perceptions persist. When STM and IDS are considered together, one partner's stress can activate a feedback loop, posing a threat to the other by triggering the IDS response. This situation suggests that individuals who have experienced defeat and entrapment may harm their relationships by presenting them as a threat to their companions, in addition to their own negative experiences. Certainly, tension is a component of the lives of all individuals. Consequently, it is crucial for partners to possess adaptive coping strategies.

Mindfulness is gaining popularity as a stress-management technique [10]. According to Skelly & Estrada-Chichon [11], using this technique as a strategy to manage anxiety and stress also yields positive outcomes as a relaxation technique. A variety of meditation exercises typically teach mindfulness, which involves deliberately focusing one's attention on the current internal and external experiences [12]. Moscardini [13] recognizes mindfulness as a detrimental predictor of entrapment. This demonstrates that individuals who are mindful experience less entrapment. Griffiths et al. [14] define entrapment as the perception of being unable to depart from undesirable circumstances and encountering a negative experience. Choi and Shin [15] have established that cognitive control serves as a moderator and hopelessness serves as a mediator in the relationship between entrapment and depression. Researchers simultaneously discovered a link between entrapment and an increased incidence of suicidal behavior and negative mental health [16]. Systematic review studies have demonstrated the efficacy of mindfulness-based interventions on suicidal behavior and well-being [17, 18]. Manzour et al. [19] found a positive correlation between entrapment and high levels of perceived stress. Additionally, engagement in a mindfulness-based stress reduction program results in substantial reductions in perceived stress and substantial enhancements in mindfulness abilities [20]. Studies that employ both longitudinal and dyadic methods elucidate the relationship between mindfulness and relationship quality, perceived partner responsiveness, attachment, empathy, and relationship preoccupation [21, 22]. Studies clearly discuss mindfulness in contexts such as romantic relationships and marriage. The Mindfulness-Based Stress Reduction intervention enhances marital satisfaction, relationships, and conflict resolution [23]. Additionally, Molajafar et al. [24] recognize that mindfulness and emotion regulation training mitigate marital disputes. On the basis of these, it is clear that it is crucial for married couples to be mindful in order to forge a strong connection, effectively manage emotional challenges, and resolve disputes in a constructive manner.

### The Present Study

According to STM, the diagnosis of a severe illness, disability, or mental disorder in one companion also impacts the other partner. Consequently, it is necessary to address the stressor in a bilateral and individual manner [25]. Simultaneously, the resources of one partner augment the resources of the other, thereby generating new synergies [8]. Consequently, they possess a greater number of resources to manage their situation. From this standpoint, the present investigation addresses a critical lacuna in the literature by investigating the connections between the concepts of entrapment, mindfulness, and mental well-being in married individuals. Despite discrete studies examining these concepts, no prior research has examined their interaction among married couples. This research addresses a critical issue that is crucial for spouses to maintain their mental health and achieve a higher level of well-being. The function of mindfulness in marriage between mental health and the reflections of the feeling of entrapment experienced by individuals on the relationship as a negative experience is also examined. The hypotheses that will be investigated within the current research have been determined in light of all of the aforementioned.

- *RQ1*. In the context of actor effect, does mindfulness in marriage play a mediating role in the relationship between entrapment and mental well-being in female and male?
- *RQ2*. In the context of partner effect, does mindfulness in marriage have a mediating role in the relationship between entrapment and mental well-being in couples?

## Methods

### Participants and Procedure

The survey involved a total of 423 heterosexual married couples in Turkey, which accounted for 846 individuals. The mean age of the female participants was 35.80 years (SD = 9.57), while the mean age of the male participants was 39.0 years (SD = 10.46). 49.4% of these couples had been together for over a decade. Among these married couples, 27.7% have no children, 27.7% have only one child, 31.0% have two children, and 19.3% have at least three children. At least one juvenile was present in 72.3% of the cases. 79.5% of women and 80.4% of men completed at least high school. Social media was employed to gather participant information. The investigation required participants to have married status. All participants in the investigation provided informed assent. This investigation adhered to the principles of the 1975 Declaration of Helsinki, which underwent revision in 2000. The study protocol has been approved by the Yıldız Technical University Scientific Research and Ethical Review Board (Address: ‘etik.yildiz.edu.tr/dogrula ’Report No: 20250104185 Verification Code: e8151).

### Measures

#### Warwick-Edinburgh Mental Well-being Scale Short Form

Tennant et al. [5] devised the scale to measure mental well-being, which is one of the fundamental concepts of positive psychology. Demirtaş and Baytemir [26] adapted the scale to Turkish. The scale, which participants completed by assessing their experiences over the past two weeks, indicates a high level of mental well-being with high scores. Cronbach’s Alpha reliability value calculated for this scale in the current study is 0.85. The seven-item scale, which is appropriate for adult samples, is composed of positive statements in a five-point Likert-type structure (1 = Never, 2 = Rarely, 3 = Sometimes, 4 = Often, 5 = Always). The scale's fit values were determined to be RMSEA = 0.065; SRMR = 0.040; CFI = 0.99; NFI = 0.97; GFI = 0.96; AGFI = 0.91.

#### Entrapment Scale Short Form

Türk et al. [27] adapted the scale into Turkish, and the Cronbach's alpha coefficient is 0.88. In the current study, this value was calculated as 0.85. De Beurs et al. [28] conducted the scale's development study, which established its position in the literature. A five-point Likert scale evaluates the responses, with answers ranging from 0 (not at all suitable for me) to 4 (completely suitable for me) and four items. The scale has a single dimension. Individuals experience a greater sense of entrapment as their score on the scale rises, a trend that is consistent with adult samples.

#### Mindfulness in Marriage Scale

Erus and Deniz [29] devised this scale to assess the degree of interpersonal mindfulness in marital relationships. The Cronbach's alpha internal consistency coefficients were 0.87 and 0.85, as determined by the calculations. Cronbach’s alpha values calculated in the current study are 0.80 and 0.83. The scale displays a single dimension and contains 12 elements. The fit values were determined to be RMSEA = 0.04, SRMR = 0.05, GFI = 0.93, CFI = 0.99, NFI = 0.95, AGFI = 0.90, and NNFI = 0.98, indicating that the scale demonstrated a satisfactory fit. The response options are a five-point Likert scale, with 1 representing never, 2 representing rarely, 3 representing sometimes, 4 representing most of the time, and 5 representing always. The scale has a maximum value of 60 and a minimum score of 12. The scale suggests that a high score indicates a high level of interpersonal mindfulness in the individual's communication and relationship with their spouse.

### Data Analysis

In this study, the predictability of mental well-being in married couples by entrapment through mindfulness was examined. A reciprocal-relational model was used in which both direct and indirect effects of couples on each other and their effects on themselves were tested.

Initially, the data from the pairings was matched, and the final data set was derived in this manner. IBM SPSS Statistics 23 was employed to conduct preliminary analyses, which included reliability, descriptive, and correlational analyses (Table 1). Afterward, the structural model was tested using AMOS using binary analysis techniques performed through path analysis. The analysis included the implementation of the item-parceling technique [30] in single-factor assessments. The unidimensionality of mental well-being and meaning-based coping in SEM led to the implementation of item-parceling. Parceling is the process of grouping individual elements into one or more"parcels"and using these parcels as signs of the target latent construct instead of individual items [31, 32]. In concepts associated with personality characteristics, the parcellation method is employed to decrease the number of observed variables, enhance reliability, and facilitate the display of a normal distribution on the scales [33]. In order to assess the overall fit of the data, a variety of fit indices, including Goodness of Fit Index (GFI), Comparative Fit Index (CFI), Tucker-Lewis Index (TLI), Incremental Fit Index (IFI), Normed Fit Index (NFI), and Standardized Root Mean Square Residual (SRMR), were applied [34]. APIM, a binary analysis approach, and the Actor-Partner Interaction Mediation Model (APIMeM), an extension of APIM, were implemented. Finally, the indirect effects of the relationships between the variables were investigated using the bias-corrected bootstrapping procedure in 5000 resamples and a 95% confidence interval.

**Table 1:** Correlations of all variables for male (above diagonal) and female (below diagonal)

Variable	Mental well-being	Entrapment	Mindfulness
Mental well-being	–	-.48	.51**
Entrapment	-.48**	–	-.45**
Mindfulness	.42**	-.42**	–
**p <.001			

## Results

### Preliminary Analysis

Table 1 illustrates the correlation relationships among the variables in the study. Table 2 displays descriptive statistics. The correlations among the variables indicated that both women's and men's mental well-being and marital mindfulness were positively correlated (Female *r* = 0.28, *p* < 0.01; Male r = 0.51, p < 0.01), while entrapment exhibited a negative correlation with mental well-being (Female *r* = −0.48, *p* < 0.01; Male *r* = −0.48, *p* < 0.01) and marital mindfulness (Female *r* = −0.42, *p* < 0.01; Male *r* = −0.45, *p* < 0.01).

**Table 2:** Descriptive statistics for the study variables

Variable	Mean	SD	Skewness	Kurtosis	McDonald ω	Cronbach α	Guttmann λ6
Mental well-being (Female)	28.09	4.91	-.569	.169	.848	.845	.837
Mental well-being (Male)	28.72	5.11	-.645	-.300	.855	.852	.848
Entrapment (Female)	4.56	4.30	.741	-.358	.848	.848	.871
Entrapment (Male)	3.37	4.08	.989	.168	.852	.851	.830
Mindfulness (Female)	51.23	6.61	-.824	-.119	.789	.801	.836
Mindfulness (Male)	50.83	7.30	-.836	.120	.836	.835	.857

### Actor-Partner Independence Mediator Model (APIMeM)

**Fig. 1:**
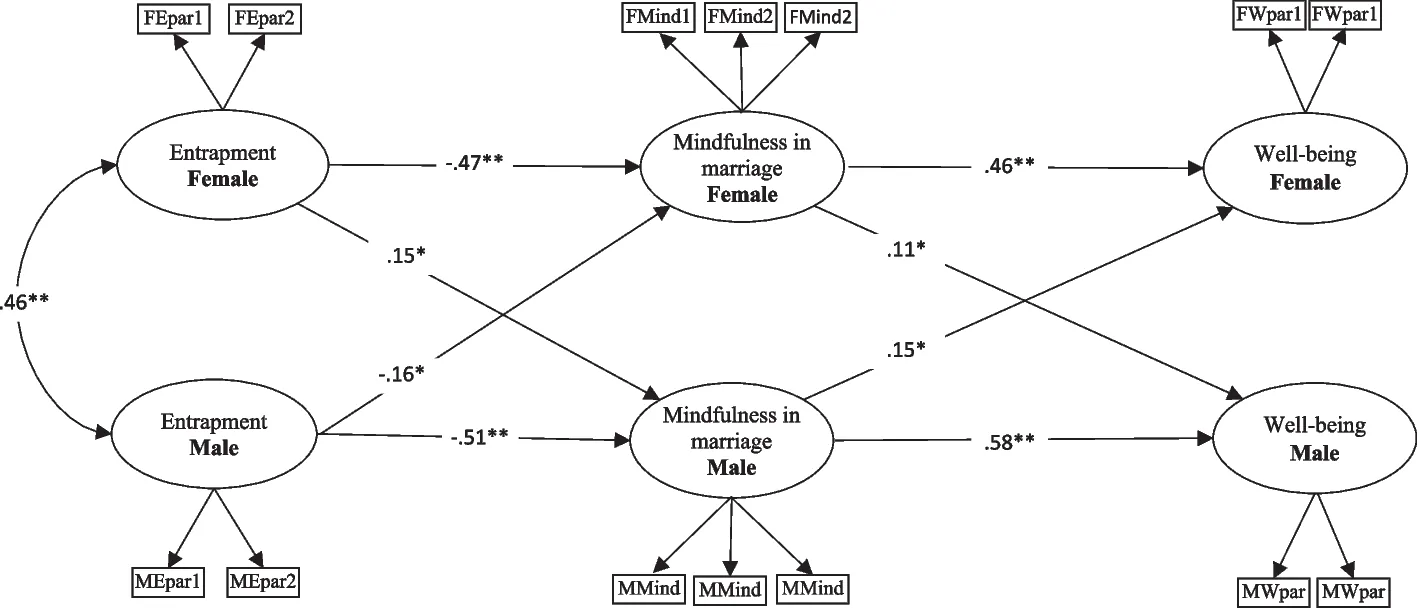
Actor-Partner Independence Mediator Model. *Note.* *N = *846*;* ^*^ *p* <.05, ^**^ *p* <.01; FEpar parcels of female’s entrapment; MEpar parcels of male’s entrapment; FMind parcels of female’s mindfulness in marriage; MMind parcels of male’s mindfulness in marriage; FWpar parcels of female’s well-being; MMpar parcels of male’s well-being

After the preliminary analyses were finished, the actor-partner interdependence mediation model was employed to evaluate the mediating role of mindfulness in the relationship between entrapment and mental well-being in married couples. The results indicated that the mediation model's fit index values were satisfactory: χ^2^/df = 3.622, *p* < 0.001; CFI = 0.943, TLI = 0.924, GFI = 0.922, NFI = 0.924, IFI = 0.944, SRMR = 0.077, RMSEA = 0.079. Additionally, the path coefficients for the actor effects between entrapment, mindfulness, and mental well-being were significant for both men and women (*β* = −0.466, *p* < 0.001 and 0.463, *p* < 0.001 for women and *β* = −0.515, p < 0.001 and 0.582, *p* < 0.001 for men, respectively) (refer to) Fig. 1. Additionally, the path from women's entrapment to men's mindfulness (*β* = −0.146, *p* < 0.05) and from men's mindfulness to women's mental well-being (*β* = 0.149, *p* < 0.05) were statistically significant in terms of partner effects. In addition, the path from men's entrapment to women's mindfulness (*β* = −0.155, *p* < 0.05) and from women's mindfulness to men's mental well-being (*β* = 0.129, *p* < 0.01) was also statistically significant.

Ultimately, bootstrapping analyses were implemented to investigate indirect effects associated with partner effects. The initial indirect effect discovery was that the mental well-being of women was indirectly influenced by men's mindfulness, as evidenced by the bootstrap value of −0.133 (95% CI [−0.205—−0.072]). The second indirect effect discovery was that the mental well-being of men was indirectly influenced by women's mindfulness, as evidenced by the bootstrap value of −0.137 (95% CI [−0.227—−0.072]). These results indicate that both partner effects are statistically significant. The actor and partner bootstrapping table is shown in Table 3.

**Table 3:** Indirect effects on male–female relationship

		Coefficient 95% Confidence Interval		
		LL UL		
**Indirect effects**				
*Actor effects*				
Entrapment—> Mindfulness—> Mental well-being (male actor effect)		-.316	-.419	-.221
Entrapment—> Mindfulness—> Mental well-being (female actor effect)		-.238	-.345	-.147
*Partner effects*				
Male’ entrapment—> Female’ mindfulness—> Male’ mental well-being (partner effect)		-.137	-.227	-.072
Female’ entrapment—> Male’ mindfulness—> Female’ mental well-being (partner effect)		-.133	-.205	-.072

## Discussion

Individuals can address their issues through various approaches, leading to happier outcomes. The foundation of this endeavor is the conviction that family contexts can facilitate individual development, with partners playing a crucial role in mutual support. Despite the bio-psycho-social model's consideration of all three factors, social factors other than social support are often overlooked. Despite the recognition by some experts of the correlation between social stressors like divorce, loss, or negative relationships and health, interventions often overlook the social component, focusing solely on the individual [24]. As a result of identifying this deficiency, the current study investigated the contributions of entrapment, a stress factor in the individual dimension, and mindfulness, a coping mechanism, to well-being within the context of partners'relationships with each other. The study's dyadic design disclosed the impact of the spouses on each other's mindfulness and well-being. The present investigation aimed to explore how mindfulness in marriage mediates the relationship between entrapment and mental well-being, specifically focusing on the actor-partner relationship. Despite the existence of studies that address these concepts in the literature, the absence of dyadic studies that address these concepts indicates a significant deficiency. The dyadic approach employed in the current study, which was conducted with married couples, makes significant contributions to the literature at this juncture. The relevant literature is the context in which the findings obtained within the scope of the research are discussed.

The first hypothesis was tested, and it was found that the relationship between entrapment and mental well-being is mediated by mindfulness in marriage as an actor effect. This outcome is noteworthy for both genders, according to analyses. Research has not examined the related concepts in conjunction, but distinct studies have examined them in pairs. Researchers recognize entrapment as a factor in the development of more detrimental mental health [16]. Research has demonstrated that Mindfulness-Based Cognitive Therapy reduces the perception of entrapment and other detrimental psychological states, while also enhancing mindfulness [35]. People widely recognize mindfulness as a negative predictor of entrapment [13]. Consequently, it is anticipated that individuals who exhibit high levels of mindfulness will experience reduced levels of entrapment. Despite the lack of research on mindfulness and entrapment, independent studies have linked the two concepts. Researchers discovered a significant correlation between entrapment and depression and well-being [36]. Furthermore, a study indicates that a higher level of mindfulness is linked to a higher level of well-being [37, 38]. Furthermore, research consistently links both mindfulness and entrapment to anxiety and depression [39, 40]. The six-year follow-up of the mindfulness-based course reported an increase in well-being, mindfulness, and problem-focused coping tendencies among those who received mindfulness training, while avoidance-focused coping tendencies significantly decreased. Furthermore, de Vibe et al. [41] predicted that an increase in problem-focused coping would lead to an increase in well-being. All of these may indicate that mindfulness is effective in coping with entrapment in both women and men, leading to higher well-being.

When the literature examines the significance of the relationships between these concepts for both genders, a comprehensive study with participants from six countries reveals that men experience a more general entrapment in various aspects of life, while women tend to experience a more focused entrapment in expressions of intense emotional burden [42]. Alispahic and Hasanbegovic-Anic [43] observed that there were disparities between men and women in the subdimensions of mindfulness. Despite the fact that women scored higher on the pretest, mindfulness-based cognitive therapy was found to be effective in reducing gender differences in well-being when applied to both men and women [44]. All of these studies suggest that the current study's findings are significant for both men and women, highlighting more specific and small-scale differences rather than significant differences between genders.

The research also examined a hypothesis about partner relationships. The analysis has revealed that men's mindfulness in marriage serves as a mediator between the mental well-being of women and entrapment. Similarly, women's mindfulness in marriage mediates between men's entrapment and men's mental well-being. In other words, the research results suggest that the entrapment of both women and men in marriage can forecast a decrease in mindfulness in their spouses, which in turn can predict a negative state of their own mental well-being. In a dyadic study, Zhang et al. [45] hypothesize that mindfulness may have contributed to relationship maintenance by lowering stress levels for both the individual and their partner. This study contributes to the understanding of the connections between mindfulness and entrapment in married couples, as entrapment is a detrimental circumstance for individuals and is positively associated with perceived stress [19]. Interpersonal mindfulness significantly positively correlates with the life satisfaction levels of married individuals [46]. Again, a study with married couples found that emotional intelligence and marital adjustment partially mediate the relationship between interpersonal mindfulness in marriage and well-being [47]. Furthermore, Cankurtaran et al. [48] anticipate that married individuals who practice mindfulness will experience fewer marital conflicts and higher marital satisfaction. In a dyadic study, APIMs demonstrated that men and women who were more mindful reported higher levels of life satisfaction, parenting warmth, and positive family expressivity, as well as lower levels of parental hostility and negative family expressivity [49]. This corroborates the current research finding by demonstrating the contributions of mindfulness to family structure within the context of the actor and partner effect. Another study found a positive and significant correlation between mindfulness levels, marital problem solving, and marital satisfaction levels, with marital problem solving predicting marital satisfaction [50]. Based on all these findings, it is possible to view married individuals experiencing entrapment as a negative indicator for their own well-being, as it may relate to their spouses becoming less mindful. However, it is also possible to view this situation as a positive indicator for their own well-being, as it may lead to their spouses being more mindful.

### Limitations

The current research has some limitations, in addition to making significant contributions to the literature. First, it's crucial to acknowledge that the study employed a dyadic approach and a cross-sectional design. Strong inferences regarding the causal relationship between concepts may not be feasible due to the cross-sectional design. To prevent this, it may be advisable to prioritize experimental studies with married couples and conduct both dyadic and longitudinal studies. Secondly, the potential consequences of social desirability and biased responses must be considered, as the data were collected through self-report from volunteer participants. Future research could use peer review, experimental design, or observation to measure factors. Furthermore, as the participants were exclusively Turkish, it would be beneficial to assess the findings for various populations to ensure that the results are applicable to various cultures. Lastly, only mindfulness was used in this study to investigate the connection between entrapment and mental well-being. However, this model does not take into account other possible mediating factors that could influence the dyadic structure, such as empathy and emotion regulation. It is suggested that future research look at these correlations using more intricate and multivariate structures, as this is regarded as a factor restricting the study's scope.

### Implications

This research elucidates the relationships between entrapment, mindfulness, and well-being, thereby rendering the dynamics of marital relationships more comprehensible. Consequently, it is anticipated that the findings will be advantageous to mental health professionals, particularly those who specialize in family and couple therapy. The therapeutic process may improve both partners'well-being. Negative consequences may arise for the partner's well-being if one partner is unmindful or experiences entrapment. In contrast, mindful people protect themselves and others from stress. The mindfulness-based group counseling program is known to enhance the marital mindfulness scores of married couples [51]. Furthermore, studies have recognized the beneficial impact of mindfulness-based interventions on well-being [52]. This approach may improve marriage well-being and mindfulness. Furthermore, interventions for the sense of entrapment that individuals experience can be devised, and their efficacy can be evaluated. Conversely, the current study can serve as a source of inspiration for future research, as there is a dearth of dyadic studies in the literature. Consequently, future research on the dynamics of couples can employ the same methodology to investigate the relationships between various concepts. Lastly, the family comprises not only spouses but also offspring. Consequently, it is believed that the purpose of conducting dyadic studies is to more comprehensively understand and develop the family structure by incorporating parents and their offspring for future research.

### Conclusion

This study examined the actor-partner relationships between entrapment and mental well-being using a dyadic method, which involved investigating the mediation of mindfulness. The relationships between these concepts were determined to be substantial at both the actor and partner levels. The results showed that the entrapment levels of the individuals predicted the mindfulness levels of both them and their partners. Furthermore, mindfulness levels similarly predicted the mental well-being of both the individual and their partner. Accordingly, it is expected that if the mindfulness levels of individuals experiencing entrapment are high in themselves and their partners, the well-being of both couples will be at a better level. Consequently, valuable information regarding the well-being of family structure was disseminated.

## Data Availability

Data will be available on request. The dataset is available upon reasonable request.

## Declarations

### Pre-registration Statement

This study was not pre-registered.
